# Influence of silicate on enrichment of highly productive microalgae from a mixed culture

**DOI:** 10.1007/s10811-015-0678-2

**Published:** 2015-08-11

**Authors:** Peter R. Mooij, Lisanne D. de Jongh, Mark C. M. van Loosdrecht, Robbert Kleerebezem

**Affiliations:** Department of Biotechnology, Delft University of Technology, Julianalaan 67, 2628 BC Delft, The Netherlands

**Keywords:** Mixed cultures, Selective environment, Green algae, Diatoms, Storage compounds

## Abstract

Microalgae have the potential to supply a biobased society with essential feedstocks like sugar and lipids. Besides being productive, strains used for this purpose should grow fast, be resistant to predators, and have good harvestability properties. Diatoms, a class of siliceous algae, have these and other preferred characteristics. In this paper, we describe the enrichment of microalgae in sequencing batch reactors with and without supply of silicate. Both reactors were operated with a light–dark cycle. To maximize storage compound production, carbon fixation and nitrogen uptake were uncoupled by limiting the availability of nitrate to the dark phase. After ten cycles, a stable culture was established in both reactors. The diatom *Nitzschia* sp. dominated the silicate-rich reactor, and the green algae *Chlamydomonas* sp. dominated the silicate-depleted reactor. Over the remaining 27 cycles of the experiment, the microalgal community structure did not change, indicating a highly stable system. Although the dominant microalga was highly dependent on the presence of silicate, the performance of both microalgal enrichments was similar. Polymers of glucose were stored during the nitrogen-limited light period. On organic matter dry weight basis, the sugar content of the biomass increased during the light period from 17 ± 4 to 53 ± 4 % for the silicate-limited culture, and from 14 ± 4 to 43 ± 4 % (*w w*^−1^) for the silicate excess culture. These results show that storage compound production can be achieved under various conditions, as long as a selective environment is maintained.

## Introduction

Microalgae are efficient producers of triacylglycerides (Hu et al. 2008) and starch (Markou et al. 2012) and have been proposed to supply these as feedstock for food, fuels, and chemicals to a biobased economy (Wijffels and Barbosa 2010). Nowadays, microalgal research mainly focuses on pure culture applications. Production of bulk compounds, such as triacylglycerides, at an industrial scale is however troublesome in an axenic microalgal culture (Kazamia et al. 2012; Shurin et al. 2013; McBride et al. 2014). Previously, we described an ecology-based enrichment and cultivation method which allows for stable storage compound production under nonaxenic conditions (Mooij et al. 2013, 2015). The basis of this approach is uncoupling of carbon fixation in the light and nitrogen uptake in the dark by limiting the presence of an essential growth nutrient (such as nitrate) to the dark phase. Limiting the presence of nitrate to the dark period provides a competitive advantage for storage compound-producing microalgae. Microalgae that efficiently convert CO_2_ into storage compounds in the light phase can take up the nitrate in the dark phase for biomass production, resulting in an enrichment culture consisting of efficient storage compound-producing microalgae (Mooij et al. 2013).

Despite their overwhelming abundance in nature, diatoms are largely overlooked in microalgal biofuel and biomass production research. Diatoms possess however various characteristics which make them interesting candidates for large-scale storage compound production (Hildebrand et al. 2012). These include the capacity to accumulate large amount of lipids when exposed to silicate limitation, good resistance to predators, and good harvestability. The presence of diatoms in nature is to a large extent regulated by the amount of available silicate (Werner 1977). At nonlimiting silicate levels, diatoms are effective competitors for limiting nutrients and are able to effectively utilize nutrient pulses (Litchman 2007). At external silicate concentrations exceeding 2 mM, diatoms typically represent more than 70 % of the phytoplankton community (Egge and Aksnes 1992). With increasing silicate to phosphorus ratios, diatom abundance increases in competition experiments between diatoms and nonsiliceous algae (Sommer 1985).

The first objective of this research was to investigate if selective enrichment of storage compound-producing diatoms can be established in an open system with a surplus of silicate in the medium. We expect the enriched microalgae community to differ in a system with and without silicate supply. The functionality of storage compound production is nonetheless expected to be present under both conditions, as it is a consequence of the uncoupling of carbon fixation and nitrogen uptake.

## Materials and methods

### Operating conditions

**Fig. 1:**
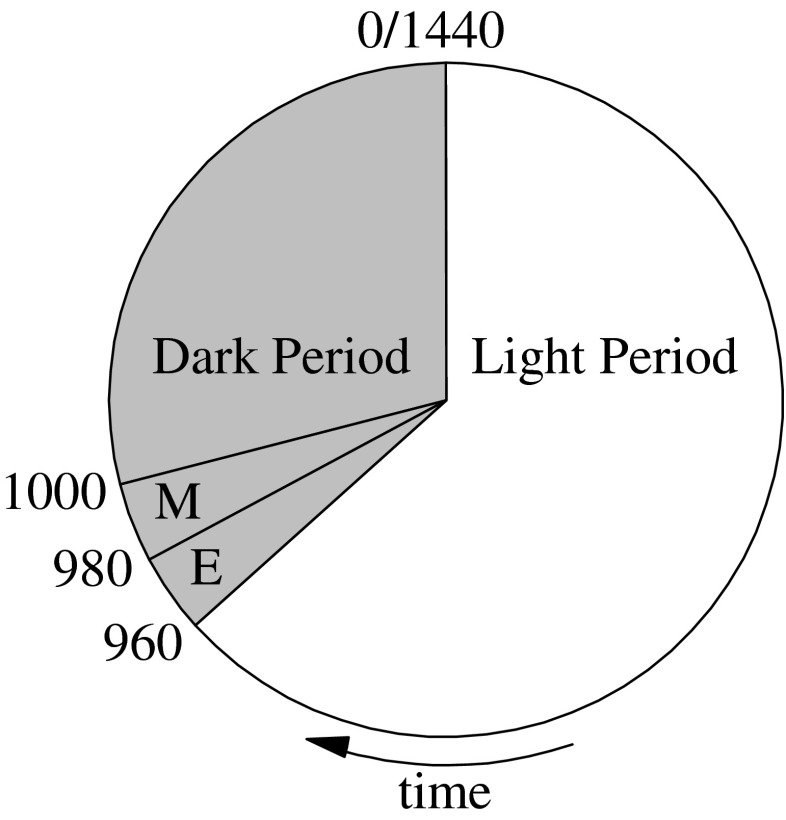
Operational cycle for reactors with effluent removal (*E*) and medium supply (*M*) at the start of the dark period. *Numbers* indicate the cumulative time in minutes from the start of the cycle

Two 1.5-L bioreactors (Applikon, The Netherlands) with a diameter of 11 cm and height of 17 cm were operated in a sequenced batch mode with the following operational parameters (Table 1). The system was operated under nonaxenic conditions. The medium composition for both reactors is described in Table 2. Na_2_SiO_3_ (11.42 mM) was supplied to the silicate-excess reactor and withheld from the silicate-depleted reactor. Nitrate was supplied as the sole nitrogen source at the beginning of the dark period and was designed to be the limiting factor for algal growth. A solid retention time of 41 h and a cycle length of 24 h (Table 1) imply that every cycle 59 % (700 mL) of the culture was harvested and replaced with medium as in Table 2. NO_3_-N (14 mg) was therefore dosed to the system at the start of every dark period. A mixture of several freshwater samples collected from the upper layer of canals, ponds, and lakes in the vicinity of Delft, the Netherlands, was used as inoculum. Figure 1 describes the operational cycle for both reactors. A Bio Controller ADI 1030 (Applikon, The Netherlands) controlled Masterflex pumps (Cole-Parmer, USA) and mass flow controllers (Brooks Instruments, The Netherlands). The Bio Controller itself was controlled by a PC with MFCS_win software (Sartorius Stedim Systems, Germany).

**Table 1:** Operational parameters

Parameter	Unit	Value
Light period	h	16
Dark period	h	8
Cycle length	h	24
Solid retention time	h	41
Liquid volume	L	1.2
Temperature	°C	28
Stirrer speed	rpm	200
Gas flow reactor in and out	NmL min^−1^	50
Gas recycle over reactor	NmL min^−1^	1200
Gas composition	% CO_2_ in air	5
Average light intensity at inner reactor surface	μmol m^−2^ s^−1^	650
Light source	–	HPS lamps
pH setpoint	–	7.5
Base	–	1.0 M NaOH
Acid	–	0.5 M HCl

**Table 2:** Medium composition

Compound	Unit	Value
NaNO_3_^−^	mM	1.43
H_3_BO_3_	mM	1.66
CaCl_2_ · 2H_2_O	mM	1.07
MgSO_4_ · 6H_2_O	mM	0.64
K_2_HPO_4_	mM	0.23
FeCl_3_ · 6H_2_O	mM	0.11
Na_2_EDTA · 2H_2_O	mM	0.11
MnCl_2_ · 4H_2_O	μM	7.71
CuSO_4_ · 5H_2_O	μM	0.03
ZnSO_4_ · 7H_2_O	μM	0.69
CoCl_2_ · 6H_2_O	μM	0.43
Na_2_MoO_4_ · 2H_2_O	μM	0.77
NaHSeO_3_	μM	0.10
NaVO_3_	μM	0.01
Allylthiourea	mg L^−1^	10

### Analytical methods

Samples were taken at the transition from dark to light and from light to dark. NO_3_^−^ was determined spectrophotometrically using Dr. Lange LCK 339 NO_3_^−^ cuvette tests (Hach Lange, Germany). Silicate was determined spectrophotometrically using Dr. Lange LCW 028 SiO_2_ cuvette tests (Hach Lange, Germany). Measurements of organic dry weight, lipids, glucose-polymers, and analysis of the microalgal community structure were done as described by Mooij et al. (2014) with the following modification. Glucose polymers were heated with 0.9 M HCl instead of 0.6 M HCl. Species succession was quantified by taking pictures using a Leica DM500B light microscope (Leica Microsystems, Germany) at × 200 magnification. These pictures were used to count and sort at least 300 microalgal cells per sample.

## Results and discussion

### Microalgal community structure

**Fig. 2:**
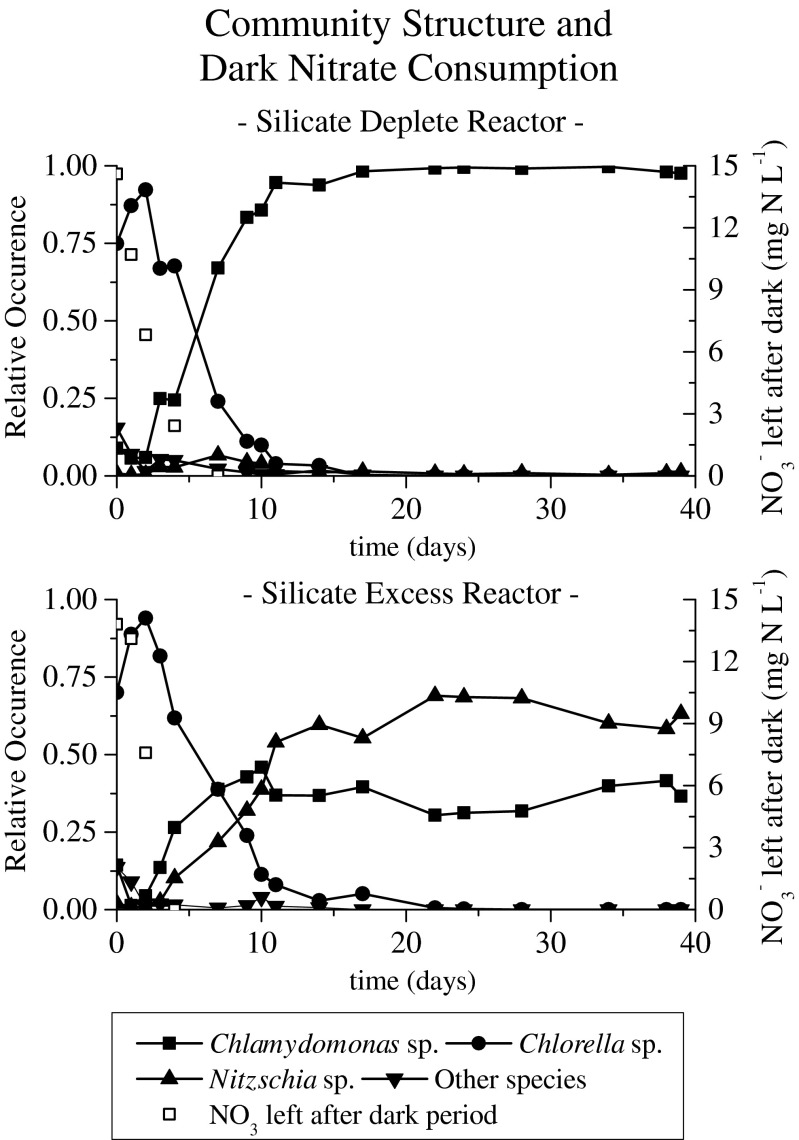
Community structure (*black lines*, *left y*-*axis*) and nitrate left over after the dark phase (*black open squares*, *right y*-*axis*) in time for the silicate-depleted (*top*) and silicate-excess (*bottom*) reactors

The first aim of this study was to investigate the influence of silicate presence on the selection of storage compound producing microalgae. In the absence of silicate, the operational conditions imposed resulted in the enrichment of the green alga *Chlamydomonas* sp. (*Lobochlamys segnis*, Table 3). With silicate in excess a coculture of the diatom *Nitzschia* sp. (accounting for 60 % of the population) and the green alga *Chlamydomonas* was established (Fig. 2). Apparently, two microalgae could coexist under the given conditions, although only one resource, nitrate, was designed to be limited. A possible explanation for the occurrence of a coculture could be a different metabolic response to the pulse-wise addition of nitrate at the start of the dark period. Diatoms are experts in nutrient uptake and storage (Litchman 2007). Nitrate storage up to an intracellular concentration of 273 mM has been reported (Kamp et al. 2011). This would allow diatoms to divide during periods without external nitrate, such as during the light period in this experiment. Nitrate storage in green algae is less documented, possibly limiting the period suitable for cell division to the dark period for green algae. This metabolic difference could possibly explain the observed coculture.

**Table 3:** Identity of species according to microscopic observation and PCR-DGGE analysis

Reactor	Species determined by microscope	Species determined by PCR-DGGE	RNA gene used	Identity (%)
Silicate excess	*Chlamydomonas* sp.	*Lobochlamys segnis* KMMCC 1045	18S	100
	*Chlorella* sp.	*Chlorella luteoviridis* CCAP 211/5B	18S	96
		*Chlorella sorokiniana* chloroplast	16S	99
	*Nitzschia* sp.	*Nitzschia frustulum* chloroplast	16S	100
Silicate deplete	*Chlamydomonas* sp.	*Lobochlamys segnis* KMMCC 1045	18S	99
	*Chlorella* sp.	*Chlorella luteoviridis* CCAP 211/5B	18S	98
		*Chlorella sorokiniana* chloroplast	16S	99
	*Nitzschia* sp.	*Nitzschia frustulum* chloroplast	16S	99

Only the main species present are depicted

The dominance of *Chlorella luteoviridis* (Table 3) increased in both reactors during the first days of the experiment (Fig. 2). After dominating both systems for around 95 % on day 3, *Chlorella* numbers steeply declined in the next days. A possible explanation lies in the different conditions before and after day 5 of operation. Nutrients were dosed at the start of every dark phase in both reactors. During the first days of the experiment, the limiting nutrient nitrate was not fully consumed in the dark phase (Fig. 2). The presence of nitrate in the light phase favored nutrient uptake and cell division over storage compound production, and this apparently facilitated *Chlorella* enrichment during this transition period. From day 5 onward, all nitrate dosed at the start of the dark phase was consumed in the dark phase; nitrate was therefore limited during the entire light phase. From this moment on, algae were not able to directly divide in the light phase. The decline of *Chlorella* sp. in the enrichment culture coincided with the onset of nitrogen limitation during the light period. Apparently, *Chlamydomonas* and *Nitzschia* outcompete *Chlorella* if carbon fixation and nitrate uptake are uncoupled. In previous work, *Chlorella luteoviridis* dominated the system throughout the experiment (Mooij et al. 2013). Operational differences between the previous and current work, such as the nitrogen source used and the solid retention time, could explain the disappearance of *C. luteoviridis* under the conditions applied in this experiment.

### Storage compound productivity in the cycle

**Fig. 3:**
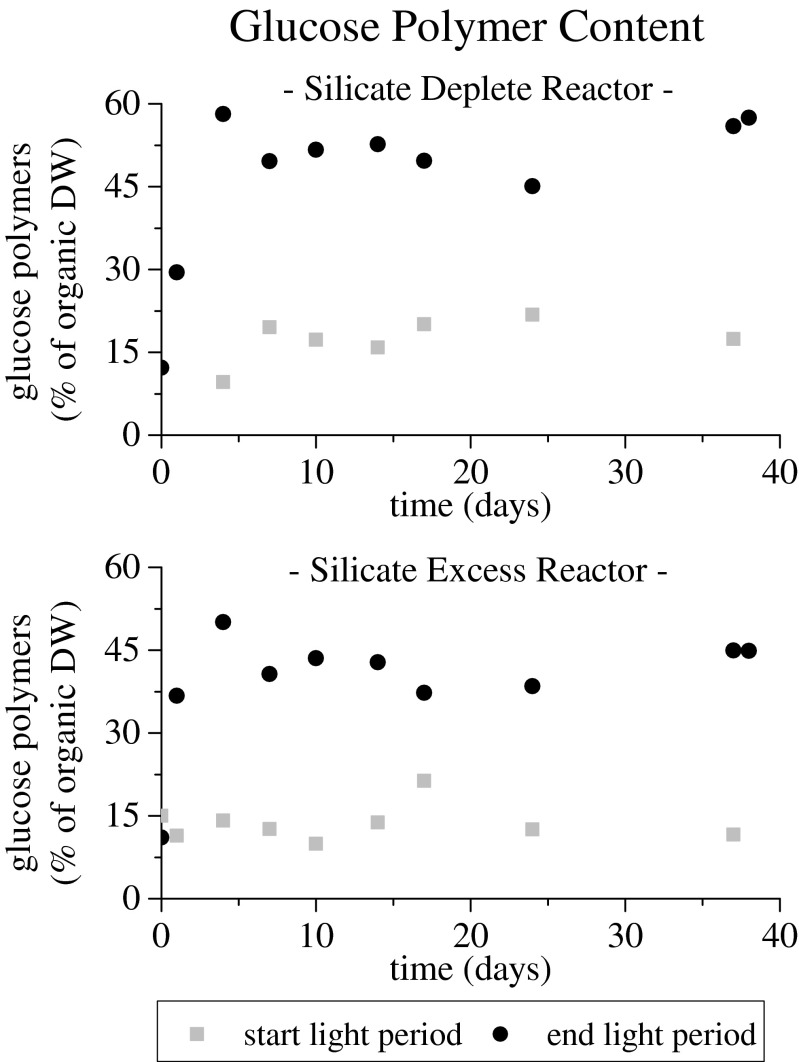
Fraction of glucose polymers on organic dry weight basis at the start (*gray squares*) and at the end (*black circles*) of the light period in time for the *silicate*-*depleted* (*top*) and silicate-excess (*bottom*) reactors

The second aim of this study concerned the functional characteristics of both systems. In both reactors, large amounts of glucose polymers were produced, increasing from 17 ± 4 to 53 ± 5 % and from 14 ± 4 to 43 ± 4 % on an organic matter basis during the light period for the silicate-depleted and silicate-excess reactors, respectively (Fig. 3). Diatoms are known to produce chrysolaminarin, a β-1,3-D-glucan, under nitrogen limitation (Granum and Myklestad 2001). Green algae produce starch under nitrogen-limited conditions (Markou et al. 2012). Both of these storage compounds will be measured as glucose monomers using our analytical methods. Lipid levels showed the same trends but were always significantly lower. The lipid fraction increased from 6 ± 1 to 7 ± 2 % and from 8 ± 2 to 10 ± 2 % on an organic matter basis during the light period for the silicate-depleted and silicate-excess reactors, respectively (data not shown). Comparing productivity values with literature values is difficult in the microalgae field. Studies differ in light input, reactor design, reactor operation, type of limitation, and other operational parameters. For pure cultures, glucose content values ranging from 41 to 62.1 % of total dry solids are reported (Dragone et al. 2011; Pirt and Pirt 1977; Brányiková et al. 2011). The values obtained for our enrichment cultures are in the same range but are reported on an organic matter basis. Due to their siliceous cell wall, the ash content of diatoms accounts for up to 50 % of the total dry solids. Comparing productivity between algae classes on a total dry solid basis should therefore be done with caution (Hildebrand et al. 2012).

### Stability

From day 10 onward, storage compound production and the microalgal population were stable in both reactors for the remaining 27 days. With around 60 % of the reactor volume harvested every day, these 27 days correspond to 30+ generations of microalgae. Gas and liquid flows leaving and entering the reactors were not sterilized, and the reactors were manually cleaned, opening the reactors fully, every 3–4 days. Despite these disturbances, the systems were very stable both in terms of functional performance and microbial community structure, emphasizing the robustness of the approach used.

## Conclusion

In the work presented here, we show that uncoupling of carbon fixation in the light and nitrogen uptake in the dark under silicate-excess conditions enriched a diatom-dominated, glucose-polymer-producing microalgae community. In the absence of silicate, a green-algae-dominated community was obtained. Both communities showed the same characteristic of producing high amounts of storage compounds in the light period and stable community structure over time. These results indicate that the proposed method will enrich in any environment a storage compound-producing algae which thrives in this specific environment. As a consequence, it allows stable storage compound production in open, and therefore cheap, cultivation systems.
